# Influence of Storage Media and Duration of Fragment in the Media on the Bond Strength of the Reattached Tooth Fragment

**DOI:** 10.5005/jp-journals-10005-1490

**Published:** 2018-04-01

**Authors:** Prashant Jalannavar, Anand Tavargeri

**Affiliations:** 1 Assistant Professor, Department of Pedodontics and Preventive Dentistry, P.M. Nadagouda Memorial Dental College and Hospital, Bagalkot Karnataka, India; 2 Professor and Head, Department of Pedodontics and Preventive Dentistry, SDM College of Dental Sciences & Hospital, Dharwad, Karnataka India

**Keywords:** Bond strength, Fracture, Reattachment, Storage media, Tooth Mousse.

## Abstract

**Introduction:**

Fracture of anterior teeth is the most frequent type of injury in the permanent dentition. Composite materials have made possible the use of adhesive materials and techniques, but storage of fragment in the media can enhance the bond strength. The purpose was to evaluate the influence of storage media and duration of the fragment in the media on the bond strength of the reattached fragment of teeth.

**Materials and methods:**

A total of 104 permanent maxillary central incisors were included. Samples were divided into four groups of 26 teeth each, further divided into eight groups of 13 teeth each and sectioned 3 mm apical to the incisal edge and stored in four storage media—tap water, artificial saliva, sodium fluoride, and Tooth Mousse at 12 and 24 hours. The bond strength was measured by universal strength testing machine.

**Results:**

Tooth Mousse showed statistically significant difference (p-value 0.001) compared with sodium fluoride, artificial saliva, and tap water when stored in both 12 and 24 hours’ duration.

**Conclusion:**

Tooth Mousse was a better storage media when compared with sodium fluoride, artificial saliva, and tap water.

**Clinical significance:**

Tooth Mousse can be considered as a best storage media for fragment reattachment.

**How to cite this article:** Jalannavar P, Tavargeri A. Influence of Storage Media and Duration of Fragment in the Media on the Bond Strength of the Reattached Tooth Fragment. Int J Clin Pediatr Dent 2018;11(2):83-88.

## INTRODUCTION

Traumatic injuries involving fracture of anterior tooth is one of the common problems among children and adolescents, which is detrimental to the esthetics and psychology of the individual. Coronal fractures of permanent incisors represent 18 to 22% of all the traumas affecting dental hard tissues; of these, 96% involve maxillary incisors.^1^

The treatment of coronal fracture is a considerable challenge for the dentist because they have to fulfill the parameters, like form and dimension, opacity, and trans-lucence of the original tooth to obtain a successful restoration.^2^ Although composite resin restoration is indicated in the management of fractured anterior teeth, reattachment is an excellent option when the fragment is available. With the development of adhesive dentistry came the concept of “fragment reattachment.”^3^ Reattachment offers the advantages of being a highly conservative technique and does not involve any kind of preparation that promotes the preservation of natural tooth structure, good esthetics, and acceptance by the patient who receives the treatment. Prognosis of the fragment reattachment depends on the firm attachment of the fragment to the tooth with strong bonding between the two segments and the tooth preparation. Various studies have been carried out using different materials and tooth preparation designs employed for the union of fractured segments.^4^

One of the factors that play an important role in the success of fragment reattachment is the type of storage of the fragment following trauma. Most of the case reports have highlighted the importance of hydrating the fractured segments.^5-7^ So, successful fragment reattachment depends on the intact retrieval of the fragment at the time of injury and adequate hydration of the fragment outside the mouth. Hydration maintains the vitality and original esthetic appearance of the tooth. The hydrophilic characteristic of adhesive systems also means that hydration acts to ensure adequate bond strength.^3^ The reattachment time can affect the bond strength of these reattached fragments because dentin moisture is also essential for achieving the high bond strength between the fragments and composite resin.^8^ Very few studies have been reported on the kinds of environments like saliva, water, or normal saline, in which patients may store fractured parts of teeth, like what should be done in case of avulsed teeth.^3^ The focus of the study was to examine the influence of different storage media and duration of the fragment in the media on the bond strength of the reattached fragment of teeth.

## MATERIALS AND METHODS

**Fig. 1:**
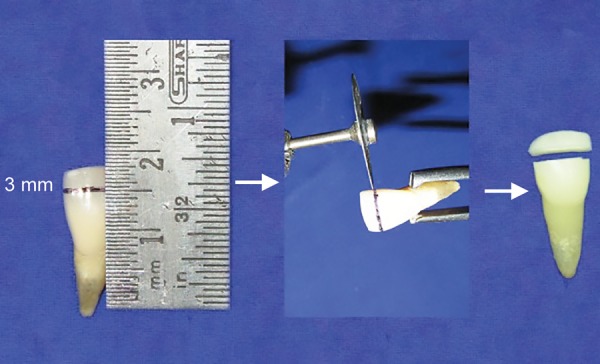
Tooth sectioned 3 mm from the incisal edge using diamond disk

**Figs 2A and B:**
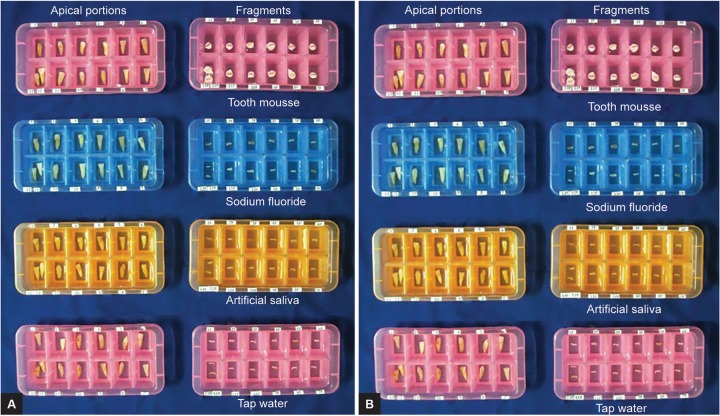
(A) Tooth fragments of the specimens in respective storage media at 12 hours. (B) tooth fragments of the specimens in respective storage media at 24 hours

In this experimental study, 104 extracted permanent maxillary central incisors were selected. All the extracted teeth were cleaned with ultrasonic scaler and samples were randomly and equally divided into four groups of 26 teeth each. Groups were further divided into eight groups of 13 teeth each (Table 1) and were marked 3 mm apical to the incisal edge. Samples were sectioned using a diamond disk (Fig. 1) and stored in already labeled respective ice trays for storage media. Four storage media were used including tap water, artificial saliva, sodium fluoride, and Tooth Mousse (Recaldent, GC Asia Dental Products, India). All the fragments were preserved in the respective storage media and the apical portions of teeth were stored in distilled water. Tap water was taken as a control group (Fig. 2).

**Table 1:** **Table 1:** Storage media for the teeth fragments according to their groups

		*Samples*							
*Storage media*		*12 hours*				*24 hours*			
Tap water		I_1_		13		A_2_		13	
Artificial saliva		II_1_		13		B_2_		13	
Sodium fluoride		III_1_		13		C_2_		13	
Tooth mousse		IV_1_		13		D_2_		13	

**Fig. 3:**
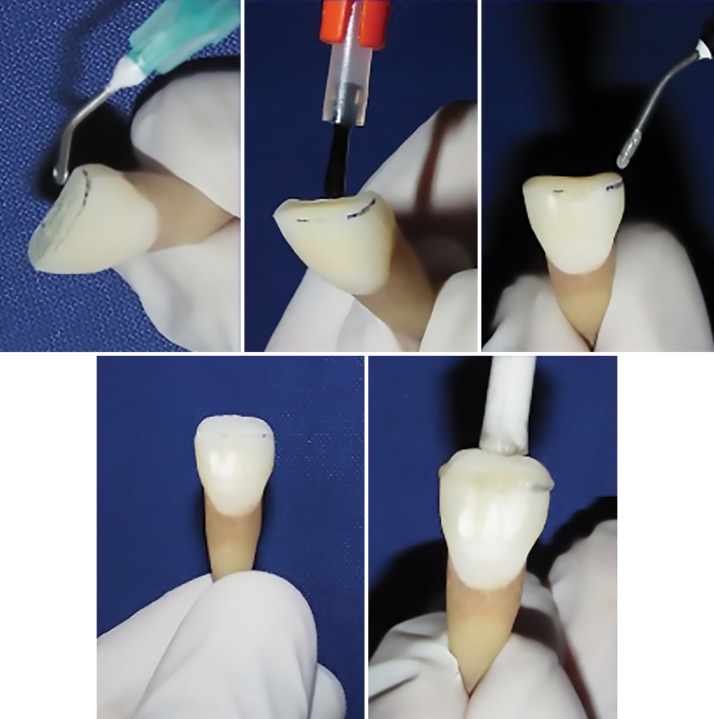
Fragment reattachment with respective tooth specimens using flowable composite

After 12 and 24 hours, in group I, fragments were rinsed, dried, and bonded to a respective apical portion of a tooth. Both the fractured surfaces were etched by 37% phosphoric acid for 15 seconds and rinsed with water for 15 seconds and dried with paper towel. Then the bonding agent was applied on both the etched surfaces and cured with light curing unit. The fragments were held with the help of gutta-percha stick and approximated with their respective apical portion using a flowable composite by pressing both the parts together and curing for 40 seconds. Reattached samples were kept in distilled water. A similar procedure was carried out respectively for groups II, III, and IV (Fig. 3).

**Fig. 4:**
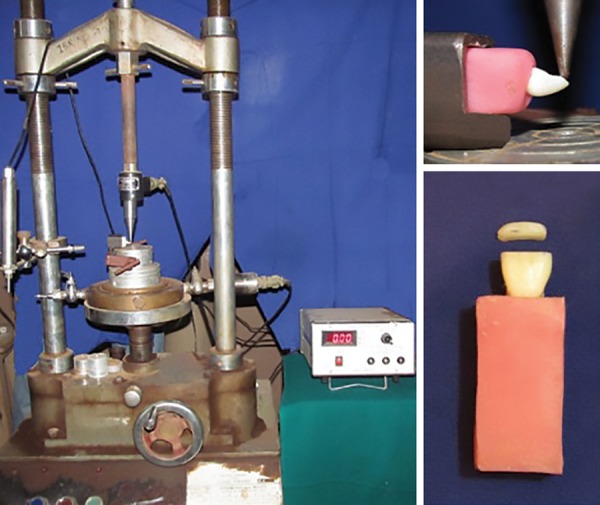
Specimens loaded on the universal strength testing machine and fractured fragment

All the samples were mounted on an acrylic block up to 1 mm apical to the cingulum and specimens were loaded on the universal strength testing machine (Instrol devices, Bengaluru, India). In order to evaluate the impact, a crosshead speed of 0.5 mm/minute was selected and the compressive load was applied on the incisal third of teeth specimen at 90° using universal strength testing machine to simulate the impact from a fall (Fig. 4). The bond strength of each specimen was tested and measured in kiloNewton and the collected data were tabulated using a Kruskal-Wallis and Mann-Whitney tests. A p-value ≤ 0.05 was considered. The statistical calculations were executed using Statistical Package for the Social Sciences version 20.0 statistical software.

## RESULTS

Kruskal-Wallis test indicated the differences in the amount of load required to fracture in different groups. There was a modest statistically significant difference between groups IV_1_ and IV_2_, and and III_2_, but the mean value of bond strength of these groups was higher than that of other groups. The mean value of bond strength was lesser in groups II_1_ and II_2_ and groups I_1_ and I_2_ at 12 and 24 hours respectively (Table 2). Mann-Whitney test was performed for intercomparison between the groups at 12 and 24 hours; groups III_2_ and IV_2_ showed statistically high significant values (p 0.001), especially groups III_1_, and IV_1_ in group III_2_ and groups I_1_, II_1_, III_1_, and IV_1 _in group IV_2_. Whereas groups I_2_ and II_2_ showed similar statistically significant values (p 0.05) when compared with the groups I_1_, II_1_, III_1_, and IV_1_ (Table 3).

**Table 2:** **Table 2:** Mean values of bond strength of reattached fragments in different storage media at 12 and 24 hours’ duration

		*12 hours storage*				*24 hours storage*			
		*Mean*		*±SD*		*Mean*		*±SD*	
Tap water		5.1785		±0.7747		7.2154		±0.7707	
Artificial saliva		6.5108		±1.0622		8.3100		±1.0029	
Sodium fluoride		12.2300		±0.9311		15.1331		±1.0896	
Tooth mousse		15.6823		±1.2168		18.7423		±1.2168	

**Table 3:** **Table 3:** Intercomparison of groups of reattached fragments at 12 and 24 hours duration and various media with respect to statistical significance

*24 hours storage*		*12 hours storage*		*Z value*		*p-value*	
Group I_2_		Group I_1_		–2.04		0.04*	
		Group II_1_		–1.9		0.05*	
		Group III_1_		–1.2		0.2	
		Group IV_1_		–1.4		0.15	
Group II_2_		Group I_1_		–1.8		0.05*	
		Group II_1_		–1.5		0.12	
		Group III_1_		–1.06		0.2	
		Group IV_1_		–0.7		0.04*	
Group III_2_		Group I_1_		–2.3		0.02*	
		Group II_1_		–2.6		0.008**	
		Group III_1_		–2.7		0.006**	
		Group IV_1_		–2.8		0.008**	
Group IV_2_		Group I_1_		–3.1		0.001**	
		Group II_1_		–3.1		0.001**	
		Group III_1_		–3.1		0.001**	
		Group IV_1_		–3.2		0.001**	
*Significant; **Highly significant							

## DISCUSSION

Maxillary central incisors were selected for the study because of the high incidence and prevalence of trauma in this region.^9,10^ Bond strength evaluation of reattached fragment is relevant because most often reattachment failures occur due to repeated trauma.^11^ Bond strength can be enhanced by storing the fragment in various storage media.

One of the factors that play a significant role in the success of fragment reattachment is the type of storage media used for the storage of fragment following trauma. If the coronal fragment has been allowed to dry out prior to reattachment, the fragment will desiccate and *in vitro* tests have shown decreased bond strength of such reat-tached fragment. Therefore, for the duration between the fragment retrieval and reattachment, the fragment should be kept moist. Storing the fragment in a moist environment ensured that there is no or minimal collapse of the collagen fibers in the dentin leading to a better bond strength. Moreover, it prevents the drying of the fragment which can be detrimental to the esthetics.^12^ Farik et al^8^ showed that drying for more than 1 hour prior to bonding of the fragment resulted in declined fracture strength.

The best storage media, as observed in the present study, was Tooth Mousse (IV_1_ and IV_2_), which had highest mean values of bond strength compared with any other experimental groups at 12 and 24 hours. On comparison within the groups, bond strength of Tooth Mousse exhibited high statistically significant difference with other groups, showing 15.68 and 18.74 kg when compared with sodium fluoride, which was 12.23 kg and 15.13 kg (III_2_) at 12 and 24 hours respectively; it could be because Tooth Mousse contains a high percentage of essential elements like calcium and phosphate. These findings were corroborating the results found by Shirani et al,^13^ where they proved that milk elements, such as calcium and phosphate can harden and stiffen both demineralized and healthy dentin by permeating the surface. This is probably the reason why enhanced bond strength was observed in the groups which were rich in calcium and phosphates. Even these results also substantiate the findings of other studies by Shirani et al,^14^ where the selection of storage conditions was based on publicly accessible materials, particularly milk and egg white, as these have been recommended for storing the avulsed tooth. Again, these media were rich in calcium and phosphate, which enhance the bond strength of reattached fragment. In addition, Borges et al^15^ also reported that fragments treated with Tooth Mousse paste prior to the application of Adper SE Plus presented statistically improved bond strength compared with nontreated samples, as confirmed by the lower frequency of adhesive failures and the higher frequency of cohesive failures in composite. Adper SE Plus presents phosphoric acid esters that are capable of bonding chemically to hydroxyapatite. Thus, this adhesion potential could have been improved by increased calcium availability after dentin treatment with Tooth Mousse paste.

In the present study, it was observed that sodium fluoride in contrast to Tooth Mousse has lower bond strength because most fluoride salts produce a reaction product which inhibits the flow of resin into spaces in and around enamel prisms, and thus decreases the mechanical retention and produces weak product-to-resin junction bond strength.^16^ It was also noted that sodium fluoride provided improved bond strength compared with artificial saliva and II_2_) of 6.51 and 8.31 kg and tap water (I_1_ and I_2_) about 5.17 and 7.21 kg; the possible reason could be the formation of fluorapatite crystals within the fragment which strengthen the reattached fragment. Moreover, artificial saliva and tap water contain minor percentage of essential elements like calcium and phosphate or fluoride; hence, they play a role in hydration of the fragment rather than the enhancement of bond strength.

One more parameter evaluated in this study was the duration of fragment in the media on the bond strength of the reattached fragment. It was observed that 24-hour groups I_2_, II_2_, III_2_, and IV_2_ demonstrated the highest bond strength mean values compared with the 12-hour groups I_1_, and IV_1_, which suggests that longer the duration of fragment in the media, higher the bond strength. These results support the findings of Farik et al.^8^

It has been reported that the strength of the bond between the fragment and tooth is reduced when the fragment is kept in a dry environment for more than 1 hour prior to its reattachment. Farik et al^8^ recommended that fragments that were initially kept in a dry environment should be kept moist (in water) for at least 24 hours prior to their reattachment. But these findings disagree with the study done by Yilmaz et al^17^ and Capp et al,^3^ where they have found that the fracture resistance of tooth fragments that were kept in a dry environment for 48 hours followed by a 30-minute rehydration were not significantly different from those teeth that had been kept in tap water for 24 hours prior to their reattachment. These data were encouraging, as a 30-minute rehydration would be clinically convenient. Hence, considering the findings of above-mentioned studies, storing the fragments for a short duration is equivalent to the 12 hours’ duration in our study.

On the contrary, in our study, it was evident that groups at 24 hours showed improved bond strength, especially Tooth Mousse group, which significantly influenced the bond strength, corroborating with Borges et al.^15^ However, in those investigations, the Tooth Mousse paste was left in place on dentin for 60 minutes per day and this application was repeated daily for 7 days. But the procedure described above to treat dentin with a Tooth Mousse paste is not clinically viable. On the contrary, this study highlights the possibility of using a shorter duration (3 minutes) to actively apply Tooth Mousse paste immediately after cavity preparation, which can be applicable for clinicians as a novel step for adhesive procedures.

Therefore, when individually considered, none of the factors (storage media and duration) were capable to restore the original strength of the teeth. However, an appropriate association between type of storage media and sufficient duration can enhance the bond strength of the reattached fragment. In this study, the appropriate association was found between Tooth Mousse and 24 hours’ duration.

Hence, the findings of above-mentioned studies support the fact that storage of the fragment in a media before reattachment would be desirable, especially calcium-rich media which can further enhance the bond strength of the reattached fragment. The lowest bond strength values were found when artificial saliva and tap water were used to store the fragment. In this way, inappropriate storage media, which have low percentage of calcium and phosphate, leads to fragility of the reattached fragment, with high risk of debonding.

## CONCLUSION

- The amount of load required to fracture the reattached fragment is influenced by the media and duration where the fragment is stored before reattachment.
- The best result is obtained when the fragment is stored in Tooth Mousse.
- Tooth Mousse can be considered as a best storage media for fragment reattachment.

## CLINICAL SIGNIFICANCE

Tooth Mousse can be considered as a best storage media for fragment reattachment, as it increases the bond strength.
